# Genetic variation and mutational determinants of azole resistance in *Candida albicans* strains of oropharyngeal colonization in HIV patients and bloodstream infections

**DOI:** 10.1186/s12929-026-01231-4

**Published:** 2026-02-22

**Authors:** Ming-Horng Tsai, Chih-Hung Hsieh, I.-An Tsai, Ching-Min Chang, Ting-Wen Chen, Jen-Fu Hsu, Shih-Ming Chu, Hsuan-Rong Huang, Shao-Hung Wang, Jang-Jih Lu

**Affiliations:** 1 Division of Neonatology and Pediatric Hematology/Oncology, Department of Pediatrics, Chang Gung Memorial Hospital, Yunlin, Taiwan; 2 College of Medicine, Chang Gung University, Taoyuan, Taiwan; 3 Institute of Bioinformatics and Systems Biology, National Yang Ming Chiao Tung University, Hsinchu, 300 Taiwan; 4 Department of Microbiology, Immunology and Biopharmaceuticals, National Chiayi University, Chiayi, 600 Taiwan; 5 Division of Pediatric Neonatology, Department of Pediatrics, Chang Gung Memorial Hospital, Taoyuan, Taiwan; 6 Division of Clinical Pathology, Taipei Tzu Chi Hospital, Buddhist Tzu Chi Medical Foundation, New Taipei, 23142 Taiwan

**Keywords:** *Candida albicans*, Antifungal susceptibility testing, Azoles resistance, *Candida* bloodstream infection, *ERG11* efflux genes

## Abstract

**Objectives:**

We aimed to analyze the genomic variations associated with high azole resistance in the *C. albicans* isolates of intensive care unit (ICU) patients with *Candida* bloodstream infections (BSIs) and those from oropharyngeal colonization in HIV patients.

**Methods:**

The genomic DNA of azole-resistant *C. albicans* isolates was analyzed using the Oxford Nanopore platform. Subsequent analyses included *ERG11* alignment to determine the extent and distribution of missense substitutions, Ka/Ks calculations to test for positive selection on *ERG11*, and detailed *CDR1* and *CDR2* mutational analysis across the coding sequence. Efflux function was assessed by measuring the fold reduction in the minimum inhibitory concentration (MIC) of azole drugs in the presence of milbemycin.

**Results:**

A total of 27 azole-resistant *C. albicans* isolates from ICU patients with *Candida* BSIs in Linkou Chang Gung Memorial Hospital in Taiwan and HIV patients’ oropharyngeal colonization were identified and analyzed. The in-depth, core analyses were performed on seven representative *C. albicans* isolates. The *C. albicans* isolates of HIV-infected patients had notably higher azole resistance (fluconazole MICs > 256 mg/L) when compared with those of the ICU patients (fluconazole MICs 16–64 mg/L), suggesting the involvement of additional mechanisms. The central role of *ERG11* mutations was supported by the presence of *ERG11* missense mutations in all azole-resistant *C. albicans* isolates. The *ERG11* coding regions analyses showed no evidence of positive selection (Ka/Ks < 1.0) and no specific mutation unique to the *C. albicans* isolates of the HIV patients, but unique missense mutations only in *CDR1*/*CDR2* were noted. Milbemycin substantially decreased the azole MICs in all *C. albicans* isolates and confirmed the efflux pump involvement. The effects of milbemycin were much lower in *C. albicans* of the HIV patients than those from the ICU patients. Furthermore, unique *ERG11* promoter variants, including changes at the Hap43p/Hap5p sites, were noted in the *C. albicans* isolates of the HIV patients.

**Conclusions:**

While *ERG11* structural mutations are foundational, the elevated azole resistance in the *C. albicans* isolates of HIV-infected patients is associated with specific *CDR1*/*CDR2* mutations and distinct *ERG11* promoter variants, highlighting genomic features that warrant further functional validation.

**Supplementary Information:**

The online version contains supplementary material available at 10.1186/s12929-026-01231-4.

## Introduction

*Candida albicans* is an important pathogen in critically ill patients, especially among those who are immunocompromised status, the intensive care unit (ICU) and human immunodeficiency virus (HIV) patients [1–3]. Invasive *C. albicans* candidiasis is associated with a high risk of treatment failure, prolonged hospitalization, infectious complications and final mortality in the era of increasing antifungal resistance [4–8]. Occurrence of antifungal tolerance and emergence of azole-resistant *C. albicans* isolates may account for the difficulties of successful eradication and unfavorable patient outcomes [9, 10].

Azole resistance of *C. albicans* develops through various mechanisms including *ERG11* mutations, which alter the ergosterol biosynthesis pathway, overexpressing of efflux pumps and altering the drug target Erg11p protein via mutations [11, 12]. Wider and more prophylactic use of antifungal agents has resulted in increased acquired resistance among clinical *C. albicans* isolates [12–14]. *ERG11* is the target gene of azoles and several mutations have been noted in the azole-resistant *C. albicans* isolates [15–17]. The *ERG11* gene encodes the lanosterol 14-α-demethylase, an enzyme interfered with azoles to inhibit the ergosterol biosynthesis, which involves the primary sterol in the fungal cell membrane, the maintenance of plasma membrane integrity and function [18, 19]. Therefore, targeting the *ERG11* gene is the major mechanism of antifungal therapies [16, 17].

Because echinocandins are large molecules, they exhibit limited penetration into certain tissues and are unsuitable for some invasive candidiasis. Therefore, azoles are not only still the first-line antifungal drugs, but also essential for prophylaxis and oral de-escalation therapy to facilitate outpatient care. Mutations in *ERG11* and azole resistance of clinical *Candida* species result in a critical global health threat that causes a higher mortality rate, may up to 100% in some cases, due to reduced effectiveness of the first-line antifungal agents [18, 20]. Increased rate of treatment failure and limitations in therapeutic options are important consequences, which would cause diagnostic challenges and more molecular techniques are needed [18, 20, 21].

Currently more than one hundred different point mutations in the *ERG11* gene have been identified from clinical *C. albicans* isolates, but only some of them were associated with azole resistance [18, 21, 22]. While azole resistance is fundamentally linked to established mechanisms—specifically *ERG11* mutations, efflux pump activity, and altered transcriptional regulation—the inconsistencies in their clinical impact highlight the significant influence of genomic diversities [17, 18], suggesting that hyper-resistance in chronic colonization niches arises from distinct promoter architecture changes and selective pressure acting in concert with these known mutations. Comparing azole-resistant *C. albicans* isolates from ICU patients and HIV-infected patients is vital, because the HIV-infected patients are often on long-term antifungal prophylaxis and profound resistance is potentially driven. Given that an increasing frequency of azole-resistant *C. albicans* isolates has been noted in the past decade [9, 13–15], we aim to investigate the genetic variations and candidate variants associated with high azole resistance in clinical *C. albicans* isolates. We hypothesized that this phenotype arises from accumulated mutations across *ERG11* coding and regulatory regions, alongside other known azole-related gene variants. These novel *ERG11* genetic diversities would potentially provide important information for development of new antifungal agents.

## Materials and methods

### Species identification

All *C. albicans* isolates were collected from pediatric and adult intensive care unit (ICU) patients with *Candida* bloodstream infections (BSIs) between 2003 and 2020 in Chang Gung Memorial Hospital (CGMH). The *C. albicans* isolates from the oropharyngeal colonization in HIV-infected patients (HIV patients) were from the National Health Research Institute in Taiwan [23, 24]. All *C. albicans* isolate identifications were performed using the Matrix-assisted laser desorption ionization time-of-flight mass spectrometry (MALDI-TOF, Bruker Biotype, software version 3.0, USA) and large-subunit (18S) ribosomal RNA gene D1/D2 domain sequencing to re-confirm all these species. This study was approved by the institutional review board of CGMH, with a waiver of informed consent because all the patient records/information were anonymized and de-identified prior to analysis.

All isolates were stored as suspensions in 40% glycerol at − 20 °C and on agar slants at room temperature until needed. Prior to testing, each isolate was passaged at least twice on SDA plates to check purity and viability.

### Antifungals and susceptibility testing

Susceptibility of all *Candida* species to nine antifungal agents was determined by broth microdilution method using a Sensititre YeastOne system (Trek Diagnostic Systems Ltd., East Grinstead, UK) according to the manufacturer’s instructions [25]. Minimum inhibitory concentration (MIC) was recorded as the highest concentration of antifungal agent resulting in the development of a blue color. All Sensititre® plates include positive control wells, and *Candida krusei* ATCC® 6258 and *Candida parapsilosis* ATCC® 22,019 were used as the quality control strains. Fluconazole, intraconazole, voriconazole, posaconazole, micafungin, caspofungin, anidulafungim, 5-flucytosine and amphotericin B were prepared according to the CLSI methods. The criteria for susceptibility of all yeast isolates to nine antifungal agents were based on MIC breakpoints of post-2014 CLSI M27-A3 and EUCAST [25, 26]. The selection of seven representative *C. albicans* isolates (comprising 2 adult ICU, 2 pediatric ICU, and 3 HIV-infected patients) from the original pool of 27 resistant strains was predicated on a 'high azole resistance' phenotype. This was specifically defined by fluconazole MICs ≥ 16 mg/L as determined via the Sensititre YeastOne system. The in-depth, core analyses were performed only on the seven azole-resistant *C. albicans* isolates.

### Whole genome sequence and similarity analyses of *Candida albicans* isolates

Genomic DNA was extracted from the *Candida albicans* cultures grown on YPD medium using a zymolyase-based protocol, followed by purification with a commercial kit, with quality assessed by Qubit fluorometry. Libraries were prepared using the Oxford Nanopore Rapid Barcoding Kit (SQK-RBK114), multiplexing up to eight samples per R10.4.1 flow cell. Sequencing was performed on a MinION Mk1C device with MinKNOW software, generating 1.20–2.38 Gb of raw data per sample and achieving a mean > 80 × across all isolates. Raw long-read sequences of the target obtained during sequencing were basecalled using the Dorado super-accuracy (SUP) model to mitigate potential basecalling inaccuracies within repetitive genomic regions. To ensure high-fidelity consensus, assemblies were aligned to the *C. albicans* SC5314 reference genome (GenBank: GCF_000182965.3); specific loci (e.g., *ERG11* and efflux pumps) and ambiguous sequences containing long mononucleotide repeats were further validated and polished using NovaSeq short-read sequencing (Illumina). De novo genome assembly was conducted with Flye version 2.9.1, resulting in 21–33 contigs with N50 values ranging from 729,298 to 1,363,968 bp. Assemblies underwent subsequent polishing and completeness assessments using BUSCO (version 5.7.0) [27, 28]. To reflect the diploid genome, missense mutations in Supplementary Tables are annotated using single-alphabet symbols for both alleles (e.g., D/D or D/E in Supplementary Tables), providing a comprehensive representation of the allelic state at each critical residue.

### Genome similarity analysis

The reference genomes of *Candida albicans* SC5314 (GenBank accession: GCF_000182965.3) and *Saccharomyces cerevisiae* S288C (GenBank accession: GCF_000146045.2) were retrieved from the NCBI RefSeq database to serve as the control and outgroup samples, respectively [29]. Average Nucleotide Identity (ANI) was calculated using the FastANI (version 1.33) method, and genomic distances was estimated using the MASH (version 2.3) method [30, 31].

### Phylogenetic analyses of *Candida albicans* isolates

#### Approach using amino acid sequences of the identified single-copy orthologs.

Universal single-copy orthologs of the *C. albicans* isolates were identified using the BUSCO and the OrthoDB dataset saccharomycetes_odb10 [27]. The *Saccharomyces cerevisiae* S288C strain was used to be an outgroup control. Concatenated amino acid sequences were aligned using the script “script_phylo.py” from the BuscoPhylo [32]. Phylogenetic trees were constructed using the Maximum Likelihood in IQ-TREE (version 2.3.6) with 1,000 bootstrap replicates. The tree visualization was performed using the Interactive Tree of Life (iTOL) web interface [33].

#### Approach using multilocus sequence typing (MLST)

The diploid sequence types (DSTs) and allele sequences from the PubMLST (schemes: *pubmlst_calbicans_isolates*, *pubmlst_calbicans_seqdef*; retrieved on May 14th, 2025) were used in the MLST approach, and the loci were identified using BLASTn. Variant-derived MLST sequences, including heterozygous loci encoded with IUPAC codes, were concatenated for phylogenetic analysis using methods consistent with the single-copy ortholog approach. Allele IDs and DSTs were assigned using the PubMLST tools. *Candida dubliniensis* CD36 (GenBank accession: GCA_000026945.1) was used as the outgroup control, which was compared with the *Candida albicans* SC5314 reference genome and isolate variants from VCF files generated by Clair3 (version v1.0.11) [34]. Allele IDs and DSTs were assigned using the online tools provided by PubMLST [35].

### Measuring selection of antimicrobial resistance-associated genes

To ensure precise genomic localization, target genes were identified via the SC5314 reference (GenBank accession: GCF_000182965.3) The key *C. albicans* genes associated with azole resistance mechanisms include *CDR1* (CGD systematic ID C3_05220W_A), *CDR2* (C3_04890W_A), *ERG11* (C5_00660C_A), *MDR1* (C6_03170C_A), *MRR1* (C3_05920W_A), *NDT80* (C2_00140W_A), *TAC1* (C5_01840C_A), and *UPC2* (C1_08460C_A). The selection of these genes was assessed using the Ka/Ks ratio [36], which was calculated from coding sequences extracted from the FASTA and GTF files of the *C. albicans* SC5314 strain genome. The source of variant data was from the VCF files generated by Clair3 [34]. Orthologous genes in *Candida dubliniensis* CD36 were identified through the BLASTn and served as references. The Ka/Ks ratios were estimated using the KaKs_Calculator2.0, which facilitates analysis of nonsynonymous versus synonymous substitution rates to infer selective pressures [27, 37].

### Characterizing the role of *CDR *effluxes on the azole resistance of *Candida albicans* isolates

Minimum inhibitory concentrations (MICs) of selected antifungals were determined using an E-test assay on Mueller–Hinton agar supplemented with 2% glucose and methylene blue (MH-GMB), with and without the efflux pump inhibitor milbemycin oxime (4 µg/mL, a concentration confirmed to be non-inhibitory for *C. albicans* growth). The E-test assays were performed in duplicate, with triplicate testing conducted if initial results were uncertain. The inoculated plates were incubated at 35 °C for 24–48 h. To ensure interpretative accuracy and reproducibility, MIC endpoints were independently evaluated by two experienced laboratory investigators. The 48-h MICs define clinical resistance, while 24-h reductions capture the kinetic magnitude of ABC transporter inhibition. A significant decrease in the MIC value in the presence of milbemycin oxime was considered a sign of efflux-mediated resistance. The inhibitory effect of milbemycin oxime was expressed as the percentage reduction in the MIC, calculated on the basis of the MIC value determined in the absence of milbemycin oxime.

### Comparison of *ERG11 *upstream regions of *Candida albicans* isolates

The nucleotide sequences of the 1000 base pairs upstream of the *ERG11* gene of all *Candida albicans* isolates were analyzed using PathoYeastrac [38]. Binding site losses were identified via PathoYeastract by comparing all seven isolates against its database of documented TF-target associations. A 100% identity consensus match within 1,000 bp upstream regions was applied to ensure high-confidence results across the cohort. The transcription factor binding sites in upstream sequences were analyzed, focusing on those linked to antimicrobial resistance.

### Data analysis and statistical methods

All statistical analyses and distribution of MIC values were performed using SPSS software (IBM SPSS Statistics version 22.0). Categorical variables were compared using the χ^2^ test, and continuous variables by the Mann–Whitney *U* test. A *P* value of 0.05 was considered significant. Resistant strains were defined according to the species-specific breakpoints proposed by the CLSI M27-S4, and EUCAST 6.1 (valid since 11 March 2013) and 5.0 (valid until 1 March 2013) documents [25, 26]. All *C. albicans* isolates showing high MICs were retested and confirmed.

## Results

### Antifungal sensitivity of clinical *Candida albicans* isolates

During the study period, a total of 27 azole-resistant *C. albicnas* isolates from adult ICU patients with *Candida* BSIs (n = 5), pediatric ICU patients with *Candida* BSIs (n = 6) and oropharyngeal colonization in HIV-infected patients (n = 16) were identified and enrolled for analyses. The MIC values of these isolates to four antifungal agents, as well as the mutation sites in *ERG11* genes and the substitution of amino acids were summarized in supplemental Table 1.

To compare the antifungal sensitivity of *C. albicans* isolates collected from oropharyngeal colonization of HIV patients and patients with *Candida* BSIs, the YeastOne panels were used for antifungal sensitivity testing. There were three *C. albicans* isolates (#5–19, #5–72, and #9–793) from the oropharyngeal colonization in HIV-infected patients, two *C. albicans* isolates (#C34 and #G01) from adult ICU patients with *Candida* BSIs, and two (#2–31 and #12–12) from pediatric patients with *Candida* BSIs were selected for further analyses. Among the seven *C. albicans* isolates, those from HIV patients exhibited profound resistance to fluconazole (> 256 mg/L) and voriconazole (> 8 mg/L). In contrast, the *C. albicans* isolates of adult ICU patients demonstrated strong resistance to fluconazole (16–64 mg/L), but lesser resistance to other antifungal agents (Table 1).

**Table 1:** The minimal inhibitory concentrations (MIC) values of the *C. albicans* isolates from adult and pediatric ICU patients with *Candida* bloodstream infections and HIV-infected patients to nine antifungal agents

MIC values (mg/L)	Echinocandins			5-FU	AB	Azoles			
Antifungal agents^a^	ANI	MIC	CAS			POS	VOC	ITC	FLC
*C. albicans* isolates									
Pediatric ICU patients									
2–31	≤ 0.015	0.015	≤ 0.015	0.5	1	**0.12**	**1**	**0.25**	**16**
12–12	≤ 0.015	0.015	0.03	1	0.5	**0.5**	**2**	**0.5**	**16**
Adult ICU patients									
C34	0.12	0.015	0.12	≤ 0.06	0.5	**1**	0.5	**0.5**	**16**
G01	0.12	0.015	0.03	0.5	1	**4**	**2**	**2**	**64**
HIV-infected patients^b^									
5–19	0.12	0.015	0.12	16	1	**0.5**	** > 8**	**0.12**	** > 256**
5–72	0.12	0.015	0.12	1	2	**16**	** > 8**	**8**	** > 256**
9–793	0.12	0.015	0.12	0.25	2	**0.25**	** > 8**	**0.12**	** > 256**

The bold colors indicate antifungal resistance

^a^Antifungal agents: ANI: anidulafungin; MIC: micafungin; CAS: caspofungin; 5-FU:5-flucytosine; AB: Amphotericin B; ITC: itraconazole; VOC: voriconazole; POS: posaconazole; FLC: fluconazole

^b^*C. albicans* from oropharyngeal colonization

### Whole genome sequence and similarity analyses of *Candida albicans* isolates

**Fig. 1:**
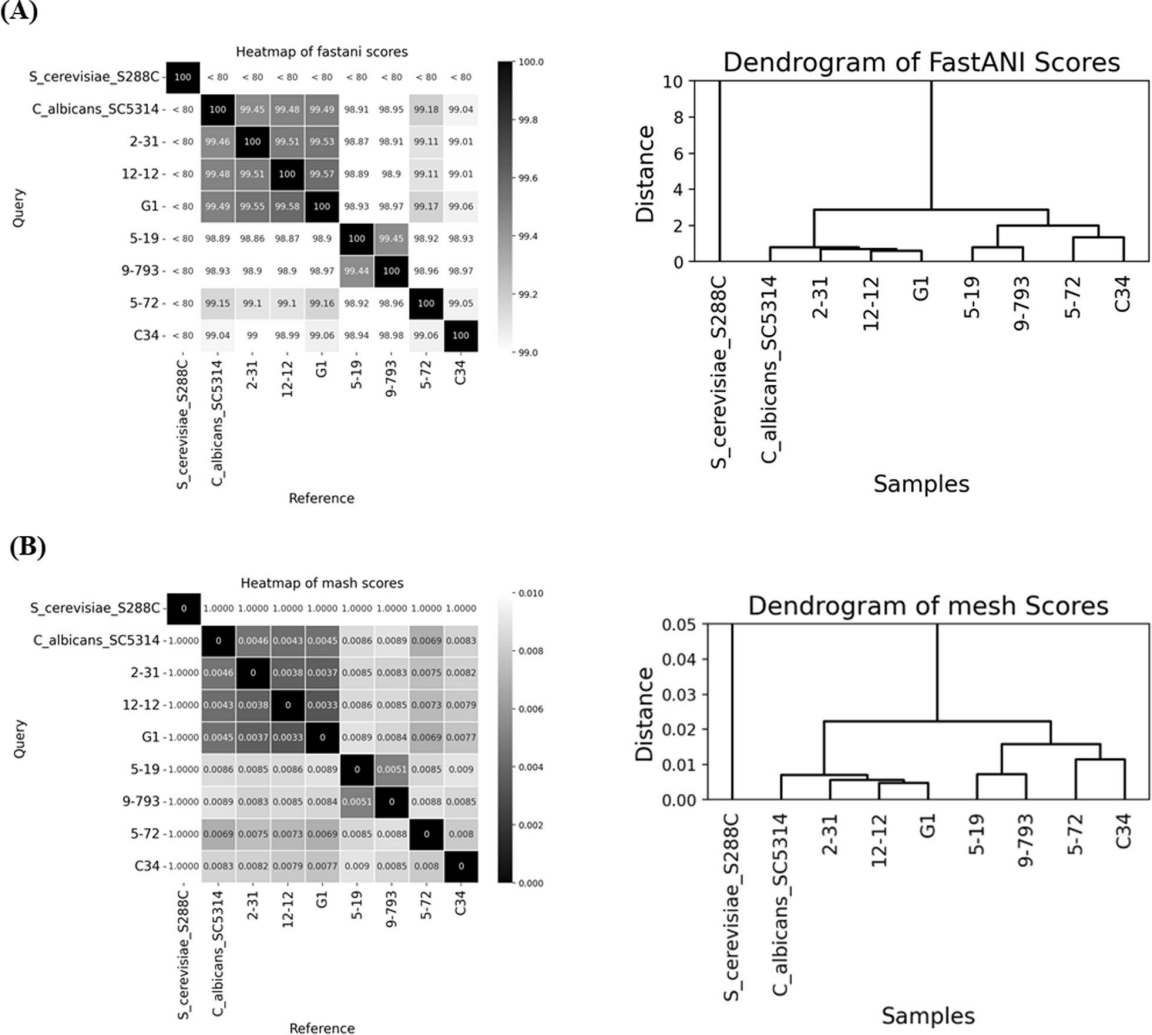
Similarity analyses of the genomes of clinical *Candida albicans* isolates and reference strains. The HIV isolates 5–19, 9–793, and 5–72 were clustered with an adult ICU isolate C34, while others grouped together, regardless of using MASH (**A**) or FastANI (**B**)

Genomic data were collected using MinION sequencing, ensuring at least 80 × coverage. The reference genomes of *C. albicans* SC5314 and *S. cerevisiae* S288C were used for phylogenetic analysis and quality control and were validated by BUSCO (Supplemental Fig. 1). The results of genomic similarity analyses using FastANI (orthologous region comparison) were compatible with those using the MASH (k-mer sketching) method (Fig. 1). The *C. albicans* #5–19, #9–793, and #5–72 formed a distinct cluster with the *C. albicans* #C34 from adult ICU patients, which revealed minimal genomic divergence. This subgroup exhibited a ≤ 0.13% ANI variation and a Mash distance of < 0.005, which indicated their near-identical genomic architectures. In contrast, the remaining isolates clustered separately, with ANI values diverging by ≥ 0.5–0.7% and MASH distances exceeding 0.005. Despite employing completely different computational approaches, both scoring systems reliably resolved phylogenetic relationships. Clustering patterns suggest potential epidemiological links or conserved genomic features (e.g., long repeat sequences or chromosomal rearrangements) among the *C. albicans* isolates from HIV patients.

### The phylogenetic analyses of clinical *Candida albicans* isolates

**Fig. 2:**
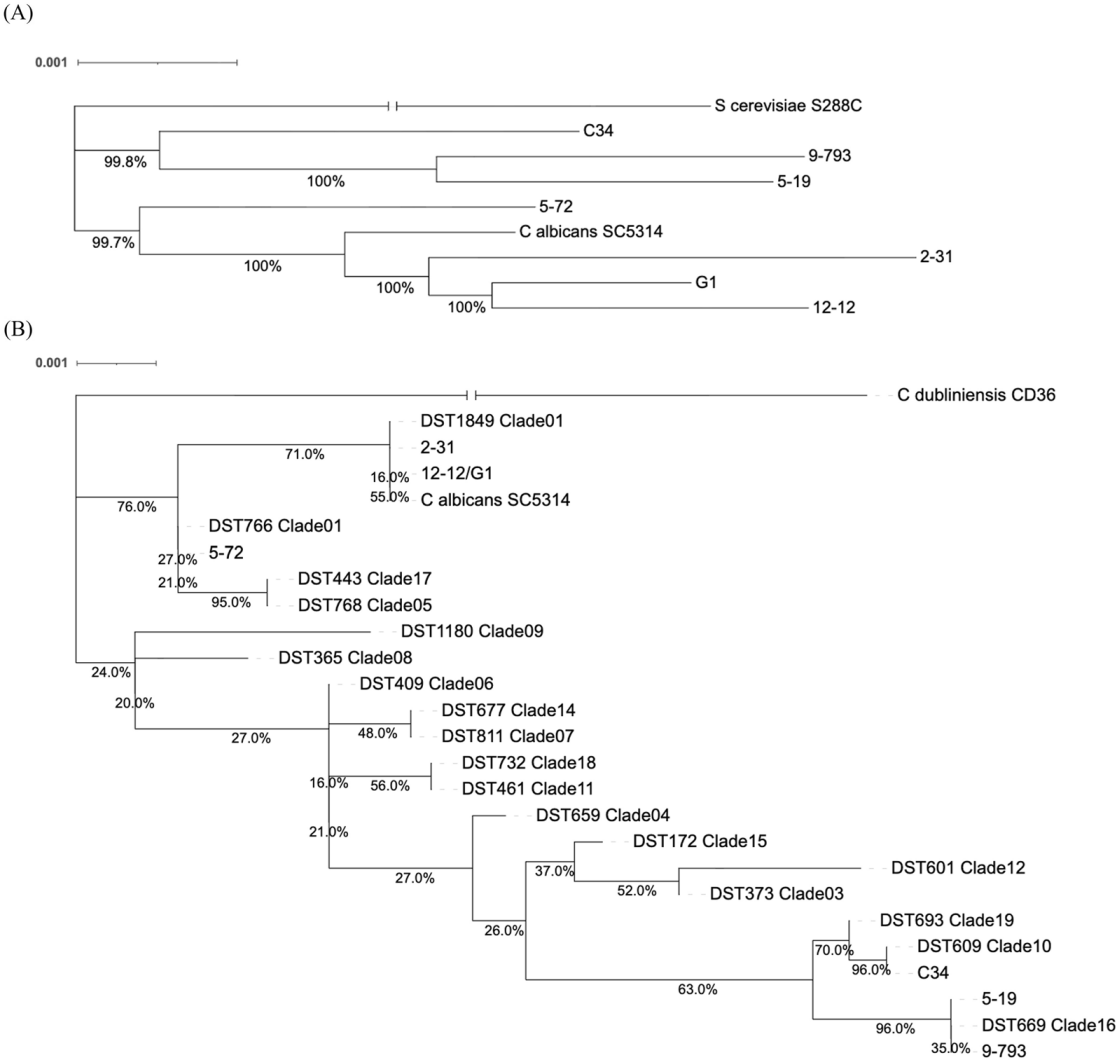
Phylogenetic analyses of clinical *Candida albicans* isolates. The whole genome sequences of the clinical isolates were compared for phylogenetic analyses, using (**A**) amino acid sequences of the identified single-copy orthologs, or (**B**) using multilocus sequence typing data. Scale bar and branch lengths indicate genetic distance (substitutions per site). Numerical labels at nodes represent bootstrap support values; branch labels indicate specific evolutionary distances

The BUSCO/saccharomycetes_odb10-identified single-copy orthologs (with *S. cerevisiae* S288C as the outgroup) and BuscoPhylo's script_phylo.py were used to complete the phylogenetic reconstruction, which showed the genomic clustering patterns among these *C. albicans* isolates (Fig. 2A). The internationally standardized MLST method, using the sequences extracted from aligned housekeeping gene alleles, was also applied for phylogenetic tree analysis with clade-representing DSTs. Both the genomic and MLST analyses revealed distinct clustering patterns among the *C. albicans* isolates, which were consistent with the above whole-genome comparisons (Fig. 2B). Of note, the *C. albicans* isolates #9–793 and #5–19, both from HIV patients, were found to cluster genomically together and align phylogenetically near the MLST clade 16. In contrast, the *C. albicans* isolates #G1, #12–12, and #2–31 from *Candida* BSIs formed a separate genomic group and corresponded to MLST clade 1.

Analysis of single-copy orthologs revealed genomic clustering, with HIV isolates #9–793 and #5–19 aligning closely, while candidemia isolates #G01, #12–12, and #2–31 grouped together. MLST data further supported this, placing #9–793 and #5–19 near clade 16 and #G01, #12–12, and #2–31 in clade 1. This result is consistent with the findings from comparing the whole genomes (Fig. 1).

### No significant nonsynonymous mutations were discovered only in *C. albicans* isolates of HIV patients

Nonsynonymous mutations were identified in the *ERG11* enzyme genes, as well as in the *CDR1*, *CDR2*, and *MDR1* efflux pump genes (Table 2, and Supplemental Table 2 & 5). The missense mutations identified in the *CDR1* and *CDR2* genes of clinical *C. albicans* isolates are summarized in Table 2. However, these mutations were not exclusively found in the *C. albicans* isolates of HIV patients with significant azole resistance.

**Table 2:** Missense mutations identified in the *CDR1* and *CDR2* genes of the *C. albicans* isolates from ICU and HIV patients

AA Range	Domain Name	Functional Description	CaCdr1p		CaCdr1p	
			ICUs	HIV	ICUs	HIV
1–80	N-terminal Domain	Regulates protein interactions and cytoskeletal associations	—		—	
81–400	NBD1 (Core)	Catalytic site for ATP binding and hydrolysis	—		121 V/V	121 V/I
401–500	NBD1-TMD1 Linker	Couples NBD1 hydrolysis to conformational changes	—		—	
501–700	TMD1	Forms translocation pore for drug and lipid transport	—		—	
701–800	Inter-domain Linker	Bridges domains to ensure structural stability	—		—	
801–1100	NBD2 (Core)	Primary energy center driving the transport cycle	842 T/T	842 S/S	1069 K/K	1069 R/R or K/R
1101–1200	NBD2-TMD2 Linker	Mediates conformational coupling between NBD2 and TMD2	—		1337 L/L or L/F	1337 F/F
1201–1450	TMD2	Determines substrate specificity and xenobiotic efflux efficiency	—		—	
1451–1499 +	C-terminal Tail	Facilitates protein folding, stability, and regulation	—		—	

Protein-coding sequences of Candida albicans (taxid: 5476) were retrieved from the NCBI nucleotide database (nt/nt) and analyzed using the tBLASTx algorithm. Low-frequency amino acid polymorphisms (less than 5%) were filtered out. Cells shaded in gray indicate mutations observed in the genome-sequenced strains of this study, while cells with a white background represent missense mutation sites obtained from the NCBI database. Two alleles coding for amino acids are separated by a slash. The IUPAC amino acid code is used to denote the substitutions. A dash ("—") indicates negligible mutations differentiating the Candida isolates between the ICU patients and HIV-infected patients. AA: amino acid; ICUs: patients from intensive care units; HIV: HIV-infected patients

The application of the efflux inhibitor milbemycin oxime consistently reduced the MICs of all *C. albicans* isolates, suggesting that these mutations may not be crucial for azole resistance (Table 3).

**Table 3:** The minimal inhibitory concentrations of the *C. albicans* isolates from adult and pediatric ICU and the HIV patients with and without the efflux pump inhibitor milbemycin oxime

*C. albicans* isolates	Fluconazole (48 h)			Voriconazole (48 h)		
	MHA	MHA + 4 μg/mL MO	Reduction (%)^$^ at 24 h	MHA	MHA + 4 μg/mL MO	Reduction (%)^$^ at 24 h
SC5314	0.38	0.19	50%	0.016	0.016	0%
2–31	8	1.5	81%	0.75	0.19	75%
12–12	2	0.25	88%	0.19	0.032	83%
C34	0.5	0.125	75%	0.032	0.016	50%
G1	8	0.19	98%	0.5	0.032	94%
5–19	0.125	0.094	25%	0.016	0.008	50%
5–72	2	0.38	81%	0.064	0.023	64%
9–793	0.38	0.19	50%	0.023	0.016	30%

MHA: Mueller–Hinton agar; MO: Milbemycin Oxime

Reduction (%) was calculated using the following formula, where positive values indicate a decrease in MIC and negative values represent an increase: ${\mathrm{Reduction}}\;{\text{(\% ) = 100}} - \left( {\frac{{{\mathrm{MIC}}\;{\mathrm{on}}\;{\mathrm{MHA}}\;{\mathrm{with}}\;{\mathrm{MO}}}}{{{\mathrm{MIC}}\;{\mathrm{on}}\;{\mathrm{MHA}}\;{\mathrm{without}}\;{\mathrm{MO}}}}\; \times \;{100}} \right)$

### Measuring selection on genes encoding antimicrobial resistance

The *ERG11* coding regions of the seven azole-resistant *C. albicans* isolates were analyzed. The observed Ka/Ks ratios were consistently below 1.0, indicating the absence of substantial selective pressure favoring amino acid substitutions in the *C. albicans* isolates from both HIV patients (all three isoltaes) and non-HIV patients (#C34) (Supplemental Fig. 2). Furthermore, the p-values for these ratios were much lower when compared with an azole-sensitive reference strain *C.* *albicans* SC5314 or unrelated *C.* *dubliniensis* CD36, highlighting the similarity in mutation trends among the *C. albicans* isolates in this study.

### Genetic variation underlying azole resistance

To understand the profound azole resistance (FLU MICs > 256 mg/L; VOR > 8 mg/L) observed in *C. albicans* isolates from HIV patients when compared to that of the ICU candidemia patients, missense mutation sites in known azole resistance-associated genes were analyzed using the NCBI tBLASTx algorithm against the nucleotide collection database (nt/nt). The genes analyzed included the ergosterol synthase *ERG11*, the efflux pumps *CDR1*, *CDR2*, and *MDR1*, as well as their corresponding regulatory genes *UPC2*, *TAC1*, *NDT80*, and *MRR1*. Numerous missense mutations were identified across all these genes; notably, missense mutations exclusive to the *C. albicans* isolates of HIV patients were found solely in the *CDR1* and *CDR2* genes (Supplementary Tables 2–5). Single-letter amino acid codes in bold denote these mutated residues, including Cdr1p at position 842 (S/S) and Cdr2p at positions 121 (V/I), 1069 (R/R or K/R), and 1337 (F/F), which suggests that efflux pumps, particularly the ATP-binding cassette (ABC) transporters, were likely contribute more meaningfully to azole resistance than other commonly known mutations.

### Examination the role of Cdr1p/Cdr2p effluxes contributing to azole resistance of *C. albicans* isolates

The contribution of efflux pumps to azole resistance was assessed by quantifying changes in fluconazole and voriconazole susceptibility in azole-resistant *C. albicans* isolates from ICU patients and HIV-infected patients. Addition of milbemycin oxime, an ABC transporter inhibitor [39], markedly enhanced susceptibility to both azoles (Table 3). Notably, ICU isolates exhibited a pronounced reduction in MICs for both drugs, whereas HIV-associated isolates showed a reduction of lesser magnitude. This differential fold decrease suggests that inhibition of Cdr1p/Cdr2p-mediated efflux contributes more substantially to azole resistance in the ICU isolates. The comparatively smaller MIC shifts in the HIV-associated isolates may indicate (i) a lower basal dependence on ABC transporter–mediated efflux, (ii) alternative resistance mechanisms (e.g., *ERG11* mutations, altered sterol homeostasis, or MFS transporter upregulation), or (iii) distinct or additional mutations in *CDR1* or *CDR2* that partially modify inhibitor binding or efflux dynamics beyond those characterized in this study. This confirms that the extreme resistance in the HIV cohort results from accumulated genetic alterations that optimize transcriptional output (e.g., the loss of Hap43p/Hap5p regulatory sites of the *ERG11* promoter) rather than reversible pump-mediated efflux alone.

### To compare the *ERG11 *upstream regions of *Candida albicans* isolates

**Fig. 3:**
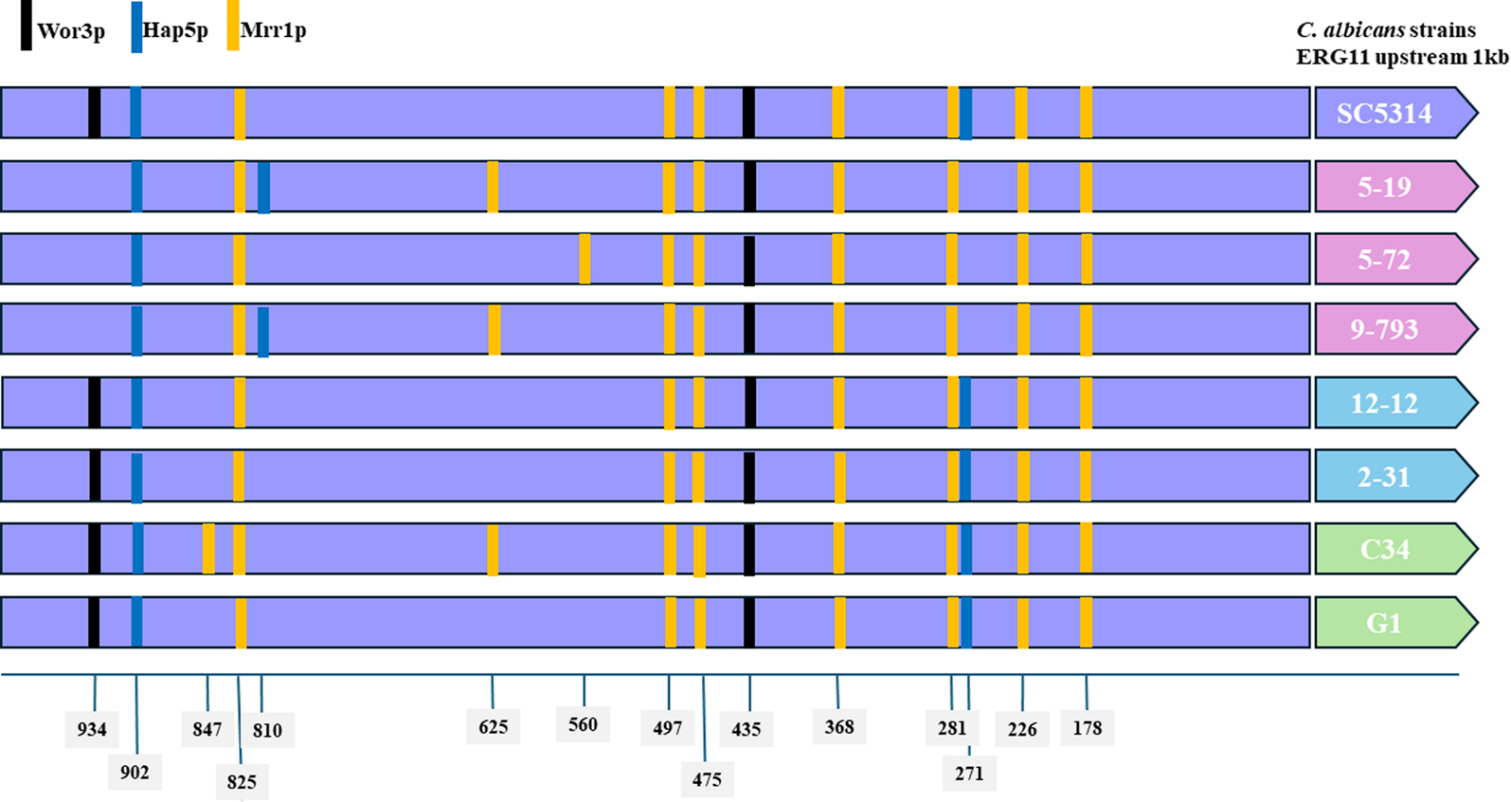
Potential transcription factor binding site mutations on *ERG11* promoters. Nucleotide sequences aligned to the *ERG11* gene of reference strain *C.* *albicans* SC5314 were retrieved and analyzed by PathoYeastrac. The variations in transcription factor binding sites corresponding to Wor3p (black vertical lines), Hap43p/Hap5p (blue vertical lines), and Mrr1p (yellow vertical lines) are indicated on the purple boxes showing the upstream 1 kb of the *ERG11* genes of HIV isolates (pink right arrows), pediatric isolates (cyan right arrows) and adult isolates (green right arrows)

To reveal the potential alteration in the upstream regulatory regions of the *ERG11* genes, 1000 base pairs of the *ERG11* coding region from the seven *C.* *albicans* isolates were retrieved and were analyzed using the PathoYeastrac [35]. Default transcription factor binding sites were applied to the sequences. Notable variations were observed within the *ERG11* promoter regions encompassing the binding motifs for Wor3p and Mrr1p transcription factors, with a significant absence of Hap43p/Hap5p (CAAT-box binding complex, CBC) binding site among the *C. albicans* isolates of HIV patients when compared to those of the adult and pediatric patients (Fig. 3). Although mutations in CBC binding have not been associated with azole resi*stance in Candida albicans, su*ch mutations have been found to reduce azole susceptibility in *Aspergillus fumigatus* [40].

## Discussion

We documented that *ERG11* gene mutations constituted the principal molecular mechanism underlying azole resistance in the clinical *C. albicans* isolates that caused BSIs in adult and pediatric ICU patients, as well as those in the oropharyngeal colonization in HIV-infected patients in Taiwan. These mutations arose without concurrent *ERG11* gene amplification, implicating specific alterations in the structural conformation of the 14α-demethylase enzyme, the primary target of azole antifungals [41]. In our cohort, the *C. albicans* isolates from HIV patients exhibited higher MICs than the isolates of other adult and pediatric patients, which suggested that further mechanisms may contribute to this variation. After analyses of missense substitutions, Ka/Ks calculations, and detailed *CDR1* and *CDR2* mutational analysis across the coding sequence, we concluded that specific *CDR1*/*CDR2* mutations and distinct *ERG11* promoter variants were associated with the elevated azole resistance in the *C. albicans* isolates of HIV-patients’ oropharyngeal colonization.

The similarity analyses and phylogenetic analyses were performed in this study in order to investigate the genetic relationships of the clinical azole-resistant *C. albicans* isolates and the representativeness of the seven isolates that were selected for further analyses. These seven *C. albicans* isolates were distributed across multiple distinct clades, rendering them well-suited for studying their genetic variation and candidate variants associated with high azole resistance. Additionally, this clade-specific grouping is consistent with prior studies showing that clade 1 is associated with clinical *C. albicans* isolates from diverse anatomical sources and its distinct clinical and genomic features, including high treatment failure and potential markers of antifungal resistance [42, 43]. The MLST data in this study confirmed the results of the genome-wide phylogenetic analysis, which further reinforces the robustness of clades in reflecting evolutionary and epidemiological relationships [42].

In the study, the ANI was calculated using FastANI, which assessed shared orthologous regions to quantify genome similarity, thus providing a high-resolution comparison of genomes among closely related *C. albicans* isolates [30]. Simultaneously, we estimated genomic distances using MASH, which employs a k-mer sketch for rapid computation, making it suitable for large-scale comparative analyses [31]. Although the two methods differ, both methods yielded consistent results in this study. Notably, the *C. albicans* isolates from HIV patients showed notably higher azole resistance, suggesting that empirical azole drug treatment may have exerted selective pressure, as these drugs primarily target the 14α-demethylase encoded by the *ERG11* gene. To investigate this possibility, The Ka/Ks ratio was calculated to compare the rates of nonsynonymous mutations (Ka) and synonymous mutations (Ks), which can provide an indicator of the selection pressure acting on resistance-related genes [36]. Because neutrality or selection (positive or purifying) is concluded when *Ka*/*Ks* is equal to or unequal to 1 [36], respectively, our results do not suggest the presence of positive selection pressure.

In the study, no specific mutations in *UPC1*, the *ERG11* transcription factor gene, or *ERG3* genes could be identified in our azole-resistant *C. albicans* isolates, which emphasized the efflux pumps to be the dominant mechanism of azole resistance [11, 44]. The *ERG3* is known as the anti-azole-associated biosynthesis enzyme gene [45, 46]. We also found *CDR1/CDR2* accumulated missense mutations, particularly in the *CDR1*, which may enhance efflux activity [11, 44]. Additionally, there was no significant structural changes in the *MDR1* mutations, which hinted that *MDR1* does not substantially contribute to drug extrusion. While expression of the *ERG11* gene is markedly upregulated upon fluconazole supplementation, we found that concurrent administration of the *CDR1/2* efflux pump inhibitor milbemycin oxime can significantly inhibit *ERG11* expression and reduce the minimum inhibitory concentration (Supplemental Fig. 3 and Table 3). These findings suggest that the regulation of *ERG11* expression plays a crucial role in controlling the susceptibility of *C. albicans* to azole drugs. While auxiliary transporters like Cdr3p (ABC-type) and Flu1p (MFS-type) may support resistance during mutation accumulation, their specific roles remain unelucidated and were excluded from our primary interpretation. Therefore, antifungal prophylaxis in HIV patients would result in selection of azole-resistant mutants in *C. albicans* isolates, with mutated efflux pumps providing basal resistance, while increased ergosterol synthesis is the key mutation distinguishing them from *C. albicans* of non-HIV patients.

While the precise contribution of the CCAAT-box binding complex (CBC) to *C. albicans* azole resistance remains to be fully elucidated, the distinct absence of CBC motifs in the *ERG11* promoters of HIV isolates (Fig. 3) suggests a regulatory decoupling from canonical sterol-mediated feedback. In *Aspergillus fumigatus*, the CBC modulates azole susceptibility by repressing the expression of the sterol-sensing factor SrbA [40]; our data support a conserved mechanism in HIV-associated isolates. This is further substantiated by efflux inhibition assays (Table 3): while the inhibitor milbemycin oxime (MO) mediated a pronounced MIC reduction (up to 98%) in *C. albicans* isolates from the ICU patients, this modulatory effect was significantly attenuated in HIV isolates (e.g., 25–50% for #5–19 and #9–793). This differential response indicates that the *Candida* isolates from HIV-infected patients do not rely solely on basal efflux; rather, the synergy between unique *CDR1*/*CDR2* missense mutations and *ERG11* promoter variants potentially drives a constitutively optimized regulation of transcriptional output that is refractory to external inhibition, accounting for the elevated MICs observed in this cohort.

The transcription factors *Tac1* and *Mrr1* (regulators of *CDR1/2* and *MDR1*) exhibited single nucleotide polymorphisms but were not associated with drug resistance levels, which highlight the importance of *ERG11* mutations, including enzyme structure and expression regulation. The main limitation of this study is the small number of cases enrolled and investigated. It is noteworthy that azole resistance is relatively low among clinically isolated *C. albicans* strains in Taiwan. Therefore, the reported mutations may not reflect global trends. Additionally, we relied on the colorimetric Sensititre YeastOne (SYO) assay, which may introduce endpoint variability compared to reference methods due to azole trailing growth. To mitigate this and ensure interpretative consistency, we strictly utilized the CLSI M27-S4 standards. We concur that future investigations should prioritize growth-based reference assessments, such as broth microdilution with optical density readings, to minimize subjective interpretation. Nonetheless, this work serves as a clinical relevant genomic study and defines the molecular basis of antifungal resistance.

## Conclusion

We found that *ERG11* structural mutations are foundational in azole resistance of clinical *C. albicans* isolates in both HIV-infected patients and ICU patients with *Candida* BSIs. We concluded that the regulation of *ERG11* expression plays a crucial role in controlling the susceptibility of *C. albicans* to azole drugs. The genomic determinants of *C. albicans* elucidated in this study establish a robust framework for clinical diagnostics and translational applications. The identification of specific transcription factor binding site variations within the *ERG11* promoter, notably the ablation of Hap43p/Hap5p (CBC) regulatory motifs, alongside unique *CDR1/CDR2* missense mutations, offers potential biomarkers for the development of targeted molecular diagnostics. These tools enable the rapid identification of highly resistant genotypes. Furthermore, regarding therapeutic interventions, the significant MIC potentiation observed with milbemycin oxime (MO) confirms the dominant contribution of ABC efflux pumps to the resistance phenotype and underscores the viability of potentiation strategies using efflux inhibitors to restore azole efficacy. Taken together, these molecular signatures facilitate a transition toward precision mycology, enabling refined diagnostic and therapeutic strategies to mitigate the burden of invasive and mucosal candidiasis.

## Supplementary Information

Additional file 1.

Additional file 2.

Additional file 3.

Additional file 4.

Additional file 5.

Additional file 6.

## Data Availability

All sequences generated in the study are publically available in the NCBI database in the following link: http://www.ncbi.nlm.nih.gov/bioproject/1423459 with the BioProject ID number: PRJNA1423459. No datasets were generated or analysed during the current study.

## References

[1] Badiee P, Boekhout T, Haddadi P, Mohammadi R, Ghadimi-Moghadam A, Soltani J, et al. Epidemiology and antifungal susceptibility of *Candida* species isolated from 10 tertiary care hospitals in Iran. Microbiol Spectr. 2022;10:e0245322. 36445122 10.1128/spectrum.02453-22 PMC9769558

[2] Kilpatrick R, Scarrow E, Hornik C, Greenberg RG. Neonatal invasive candidiasis: updates on clinical management and prevention. Lancet Child Adolesc Health. 2022;6(1):60–70. 10.1016/S2352-4642(21)00272-8. 34672994 10.1016/S2352-4642(21)00272-8

[3] Alonso-Monge R, Gresnigt MS, Román E, Hube B, Pla J. Candida albicans colonization of the gastrointestinal tract: a double-edged sword. PLoS Pathog. 2021;17(7):e1009710. 34293071 10.1371/journal.ppat.1009710 PMC8297749

[4] Alenazy H, Alghamdi A, Pinto R, Daneman N. *Candida* colonization as a predictor of invasive candidiasis in non-neutropenic ICU patients with sepsis: a systematic review and meta-analysis. Int J Infect Dis. 2021;102:357–62. 33157294 10.1016/j.ijid.2020.10.092

[5] Sprague JL, Kasper L, Hube B. From intestinal colonization to systemic infections: *Candida albicans* translocation and dissemination. Gut Microbes. 2022;14(1):2154548. 36503341 10.1080/19490976.2022.2154548 PMC9746630

[6] Soriano A, Honore PM, Puerta-Alcalde P, Garcia-Vidal C, Pagotto A, Gonçalves-Bradley DC, et al. Invasive candidiasis: current clinical challenges and unmet needs in adult populations. J Antimicrob Chemother. 2023;78(7):1569–85. 37220664 10.1093/jac/dkad139 PMC10320127

[7] Rodrigues LS, Siqueira AC, Vasconcelos TM, Ferreira AMM, Spalanzani RN, Krul D, et al. Invasive candidiasis in a pediatric tertiary hospital: Epidemiology, antifungal susceptibility, and mortality rates. Med Mycol. 2024;62(10):myae097. 39354681 10.1093/mmy/myae097 PMC11498051

[8] Thompson GR 3rd, Soriano A, Honore PM, Bassetti M, Cornely OA, Kollef M, et al. Efficacy and safety of rezafungin and caspofungin in candidaemia and invasive candidiasis: pooled data from two prospective randomised controlled trials. Lancet Infect Dis. 2024;24(3):319–28. 38008099 10.1016/S1473-3099(23)00551-0

[9] Fan F, Liu Y, Liu Y, Lv R, Sun W, Ding W, et al. *Candidaalbicans* biofilms: *antifungal* resistance, immune evasion, and emerging therapeutic strategies. Int J Antimicrob Agents. 2022;60(5–6):106673. 36103915 10.1016/j.ijantimicag.2022.106673

[10] Kilburn S, Innes G, Quinn M, Southwick K, Ostrowsky B, Greenko JA, et al. Antifungal resistance trends of candida auris clinical isolates in New York and New Jersey from 2016 to 2020. Antimicrob Agents Chemother. 2022;66(3):e0224221. 35007140 10.1128/aac.02242-21 PMC8923207

[11] Czajka KM, Venkataraman K, Brabant-Kirwan D, Santi SA, Verschoor C, Appanna VD, et al. Molecular mechanisms associated with antifungal resistance in Pathogenic *Candida* Species. Cells. 2023;12(22):2655. 37998390 10.3390/cells12222655 PMC10670235

[12] Pristov KE, Ghannoum MA. Resistance of *Candida* to azoles and echinocandins worldwide. Clin Microbiol Infect. 2019;25(7):792–8. 30965100 10.1016/j.cmi.2019.03.028

[13] Giannella M, Lanternier F, Dellière S, Groll AH, Mueller NJ, Alastruey-Izquierdo A, et al. Invasive fungal disease in the immunocompromised host: changing epidemiology, new antifungal therapies, and management challenges. Clin Microbiol Infect. 2025;31(1):29–36. 39142631 10.1016/j.cmi.2024.08.006

[14] Huang SJ, Song YH, Lv G, Liu JY, Zhao JT, Wang LL, et al. Emergence of invasive candidiasis with multiple *Candida* species exhibiting azole and echinocandin resistance. Front Microbiol. 2025;25(16):1550894. 10.3389/fmicb.2025.1550894 PMC11975943 40201445

[15] Ceballos-Garzon A, Peñuela A, Valderrama-Beltrán S, Vargas-Casanova Y, Ariza B, Parra-Giraldo CM. Emergence and circulation of azole-resistant *C. albicans*, *C. auris* and *C. parapsilosis* bloodstream isolates carrying Y132F, K143R or T220L Erg11p substitutions in Colombia. Front Cell Infect Microbiol. 2023;13:1136217. 37026059 10.3389/fcimb.2023.1136217 PMC10070958

[16] Li J, Coste AT, Liechti M, Bachmann D, Sanglard D, Lamoth F. Novel *ERG11* and *TAC1b* mutations associated with azole resistance in *Candida auris*. Antimicrob Agents Chemother. 2023;65(5):e02663-e2720. 33619054 10.1128/AAC.02663-20 PMC8092887

[17] Urbanek AK, Łapińska Z, Derkacz D, Krasowska A. The Role of *ERG11* point mutations in the resistance of *Candida albicans* to fluconazole in the presence of lactate. Pathogens. 2022;11(11):1289. 36365040 10.3390/pathogens11111289 PMC9698267

[18] Flowers SA, Colón B, Whaley SG, Schuler MA, Rogers PD. Contribution of clinically derived mutations in ERG11 to azole resistance in *Candida albicans*. Antimicrob Agents Chemother. 2015;59:450–60. 25385095 10.1128/AAC.03470-14 PMC4291385

[19] de Oliveira Santos GC, Vasconcelos CC, Lopes AJO, de Sousa Cartágenes MDS, Filho AKDB, do Nascimento FRF, et al. Candida infections and therapeutic strategies: mechanisms of action for traditional and alternative agents. Front Microbiol. 2018;3:1351. 10.3389/fmicb.2018.01351 PMC6038711 30018595

[20] Warrilow AG, Nishimoto AT, Parker JE, Price CL, Flowers SA, Kelly DE, et al. The evolution of azole resistance in *Candida albicans* Sterol 14α-demethylase (CYP51) through incremental amino acid substitutions. Antimicrob Agents Chemother. 2019;63(5):e02586-e2618. 30783005 10.1128/AAC.02586-18 PMC6496074

[21] Liu JY, Shi C, Wang Y, Li WJ, Zhao Y, Xiang MJ. Mechanisms of azole resistance in *Candida albicans* clinical isolates from Shanghai, China. Res Microbiol. 2015;166(3):153–61. 25748216 10.1016/j.resmic.2015.02.009

[22] Bédard C, Gagnon-Arsenault I, Boisvert J, Plante S, Dubé AK, Pageau A, et al. Most azole resistance mutations in the *Candida albicans* drug target confer cross-resistance without intrinsic fitness cost. Nat Microbiol. 2024;9(11):3025–40. 39379635 10.1038/s41564-024-01819-2

[23] Ho MW, Yang YL, Lin CC, Chi CY, Chen HT, Lin PC, et al. Yeast oropharyngeal colonization in human immunodeficiency virus-infected patients in central Taiwan. Mycopathologia. 2014;177(5–6):309–17. 24804977 10.1007/s11046-014-9753-5

[24] Wu CJ, Lee HC, Yang YL, Chang CM, Chen HT, Lin CC, et al. Oropharyngeal yeast colonization in HIV-infected outpatients in southern Taiwan: CD4 count, efavirenz therapy and intravenous drug use matter. Clin Microbiol Infect. 2012;18(5):485–90. 21939471 10.1111/j.1469-0691.2011.03655.x

[25] Orasch C, Marchetti O, Garbino J, Schrenzel J, Zimmerli S, Mühlethaler K, et al. Candida species distribution and antifungal susceptibility testing according to European Committee on Antimicrobial Susceptibility Testing and new vs. old Clinical and Laboratory Standards Institute clinical breakpoints: a 6-year prospective candidaemia survey from the fungal infection network of Switzerland. Clin Microbiol Infect. 2014;20:698–705. 24188136 10.1111/1469-0691.12440

[26] Clinical and Laboratory Standards Institute. Reference method for broth dilution antifungal susceptibility testing of yeasts: 4th informational supplement. Document M27-S4. Wayne, PA: CLSI; 2012.

[27] Lin B, Hui J, Mao H. Nanopore technology and its applications in gene sequencing. Biosensors. 2021;11(7):214. 34208844 10.3390/bios11070214 PMC8301755

[28] Freire B, Ladra S, Parama JR. Memory-efficient assembly using Flye. IEEE ACM Trans Comput Biol Bioinform. 2022;19(6):3564–77. 34469305 10.1109/TCBB.2021.3108843

[29] Goldfarb T, Kodali VK, Pujar S, Brover V, Robbertse B, Farrell CM, et al. NCBI RefSeq: reference sequence standards through 25 years of curation and annotation. Nucleic Acids Res. 2025;53(D1):D243–57. 39526381 10.1093/nar/gkae1038 PMC11701664

[30] Jain C, Rodriguez-R LM, Phillippy AM, Konstantinidis KT, Aluru S. High throughput ANI analysis of 90K prokaryotic genomes reveals clear species boundaries. Nat Commun. 2018;9(1):5114. 30504855 10.1038/s41467-018-07641-9 PMC6269478

[31] Ondov BD, Treangen TJ, Melsted P, Mallonee AB, Bergman NH, Koren S, et al. Mash: fast genome and metagenome distance estimation using MinHash. Genome Biol. 2016;17(1):132. 27323842 10.1186/s13059-016-0997-x PMC4915045

[32] Sahbou AE, Iraqi D, Mentag R, Khayi S. BuscoPhylo: a webserver for Busco-based phylogenomic analysis for non-specialists. Sci Rep. 2022;12(1):17352. 36253435 10.1038/s41598-022-22461-0 PMC9576783

[33] Letunic I, Bork P. Interactive Tree of Life (iTOL) v6: recent updates to the phylogenetic tree display and annotation tool. Nucleic Acids Res. 2024;52(W1):W78–82. 38613393 10.1093/nar/gkae268 PMC11223838

[34] Zheng Z, Li S, Su J, Leung AW, Lam TW, Luo R. Symphonizing pileup and full-alignment for deep learning-based long-read variant calling. Nat Comput Sci. 2022;2(12):797–803. 38177392 10.1038/s43588-022-00387-x

[35] Jolley KA, Bray JE, Maiden MCJ. Open-access bacterial population genomics: BIGSdb software, the PubMLST.org website and their applications. Wellcome Open Res. 2018;3:124. 30345391 10.12688/wellcomeopenres.14826.1 PMC6192448

[36] Li LL, Xiao Y, Wang X, He ZH, Lv YW, Hu XS. The Ka/Ks and πa /πs ratios under different models of gametophytic and sporophytic selection. Genome Biol Evol. 2023;15(8):151. 10.1093/gbe/evad151 PMC10443736 37561000

[37] Camacho C, Coulouris G, Avagyan V, Ma N, Papadopoulos J, Bealer K, et al. BLAST+: architecture and applications. BMC Bioinformatics. 2009;10:421. 20003500 10.1186/1471-2105-10-421 PMC2803857

[38] Teixeira MC, Viana R, Palma M, Oliveira J, Galocha M, Mota MN, et al. YEASTRACT+: a portal for the exploitation of global transcription regulation and metabolic model data in yeast biotechnology and pathogenesis. Nucleic Acids Res. 2023;51(D1):D785–91. 36350610 10.1093/nar/gkac1041 PMC9825512

[39] Peng Y, Lu Y, Sun H, Ma J, Li X, Han X, et al. Cryo-EM structures of Candida albicans Cdr1 reveal azole-substrate recognition and inhibitor blocking mechanisms. Nat Commun. 2024;15(1):7722. 39242571 10.1038/s41467-024-52107-w PMC11379888

[40] Zhang C, Gao L, Ren Y, Gu H, Zhang Y, Lu L. The CCAAT-binding complex mediates azole susceptibility of *Aspergillus fumigatus* by suppressing SrbA expression and cleavage. Microbiologyopen. 2021;10(6):e1249. 34964293 10.1002/mbo3.1249 PMC8608569

[41] Rosam K, Monk BC, Lackner M. Sterol 14 alpha-demethylase ligand-binding pocket-mediated acquired and intrinsic azole resistance in fungal pathogens. J Fungi. 2020;7(1):1. 10.3390/jof7010001 PMC7822023 33374996

[42] Zhu Y, Fang C, Shi Y, Shan Y, Liu X, Liang Y, et al. Candida albicans multilocus sequence typing Clade 1 contributes to the clinical phenotype of vulvovaginal candidiasis patients. Front Med. 2022;1(9):837536. 10.3389/fmed.2022.837536 PMC9010739 35433756

[43] McManus BA, Maguire R, Cashin PJ, Claffey N, Flint S, Abdulrahim MH, et al. Enrichment of multilocus sequence typing clade1 with oral *Candida albicans* isolates in patients with untreated periodontitis. J Clin Microbiol. 2012;50(10):3335–44. 22875886 10.1128/JCM.01532-12 PMC3457439

[44] Chang W, Liu J, Zhang M, Shi H, Zheng S, Jin X, et al. Efflux pump-mediated resistance to antifungal compounds can be prevented by conjugation with triphenylphosphonium cation. Nat Commun. 2018;9(1):5102. 30504815 10.1038/s41467-018-07633-9 PMC6269435

[45] Vale-Silva LA, Coste AT, Ischer F, Parker JE, Kelly SL, Pinto E, et al. Azole resistance by loss of function of the sterol delta 5,6-desaturase gene (ERG3) in *Candida albicans* does not necessarily decrease virulence. Antimicrob Agents Chemother. 2012;56(4):1960–8. 22252807 10.1128/AAC.05720-11 PMC3318373

[46] Hartuis S, Ourliac-Garnier I, Robert E, Albassier M, Duchesne L, Beaufils C, et al. Precise genome editing underlines the distinct contributions of mutations in *ERG11*, *ERG3*, *MRR1*, and *TAC1* genes to antifungal resistance in *Candida parapsilosis*. Antimicrob Agents Chemother. 2024;68(6):e0002224. 38624217 10.1128/aac.00022-24 PMC11620491

