# Supplementary material for: Genetic variation and mutational determinants of azole resistance in Candida albicans strains of oropharyngeal colonization in HIV patients and bloodstream infections
Source: J Biomed Sci. 2026 Feb 22;33:20. doi: 10.1186/s12929-026-01231-4 (PMC12925357; doi:10.1186/s12929-026-01231-4)
Supplement: Supplementary file 1 — Additional file 1. [file 12929_2026_1231_MOESM1_ESM.doc]

Supplemental Figure 1. The BUSCO assessment results of the reference strains (*C. albicans* SC5314 and *S. cerevisiae* S288C) and the azole-resistant *C. albicans*
