# Supplementary material for: Genetic variation and mutational determinants of azole resistance in Candida albicans strains of oropharyngeal colonization in HIV patients and bloodstream infections
Source: J Biomed Sci. 2026 Feb 22;33:20. doi: 10.1186/s12929-026-01231-4 (PMC12925357; doi:10.1186/s12929-026-01231-4)
Supplement: Supplementary file 2 — Additional file 2. [file 12929_2026_1231_MOESM2_ESM.doc]

|  | **Sequence** | **Method** | **Ka** | **Ks** | **Ka/Ks** | **P-Value(Fisher)** |
| --- | --- | --- | --- | --- | --- | --- |
| 0 | 12-12 | YN | 0.001433 | NaN | NaN | NaN |
| 1 | 2-31 | YN | 0.001433 | NaN | NaN | NaN |
| 2 | 5-19 | YN | 0.002228 | 0.063307 | 0.035189 | 7.994880E-10 |
| 3 | 5-72 | YN | 0.000718 | 0.043285 | 0.016583 | 3.016540E-07 |
| 4 | 9-793 | YN | 0.002228 | 0.063307 | 0.035189 | 7.994880E-10 |
| 5 | C34 | YN | 0.001440 | 0.076224 | 0.018886 | 1.007110E-11 |
| 6 | G1 | YN | 0.001433 | NaN | NaN | NaN |

(Reference *Candida albicans* SC5314)

|  | **Sequence** | **Method** | **Ka** | **Ks** | **Ka/Ks** | **P-Value(Fisher)** |
| --- | --- | --- | --- | --- | --- | --- |
| 0 | 12-12 | YN | 0.028101 | 0.705714 | 0.039819 | 3.719830E-49 |
| 1 | 2-31 | YN | 0.028152 | 0.686822 | 0.040989 | 8.968100E-49 |
| 2 | 5-19 | YN | 0.027573 | 0.653068 | 0.042221 | 3.439000E-49 |
| 3 | 5-72 | YN | 0.027474 | 0.661099 | 0.041558 | 1.833190E-48 |
| 4 | 9-793 | YN | 0.027573 | 0.653068 | 0.042221 | 3.439000E-49 |
| 5 | C34 | YN | 0.026757 | 0.696223 | 0.038432 | 4.702790E-51 |
| 6 | G1 | YN | 0.028101 | 0.705714 | 0.039819 | 3.719830E-49 |

(Reference *Candida dubliniensis* CD36)

**Supplemental Figure 2. Selection on antimicrobial resistance-associated *ERG11* genes.** The Ka/Ks ratios were used to compare missense and silent mutations in *ERG11* coding regions, indicating selection pressure on resistance genes, using reference nucleotide sequences *C. albicans* SC5314 (A) or *C. dubliniensis* CD36 (B).
