# Supplementary material for: Genetic variation and mutational determinants of azole resistance in Candida albicans strains of oropharyngeal colonization in HIV patients and bloodstream infections
Source: J Biomed Sci. 2026 Feb 22;33:20. doi: 10.1186/s12929-026-01231-4 (PMC12925357; doi:10.1186/s12929-026-01231-4)
Supplement: Supplementary file 3 — Additional file 3. [file 12929_2026_1231_MOESM3_ESM.doc]

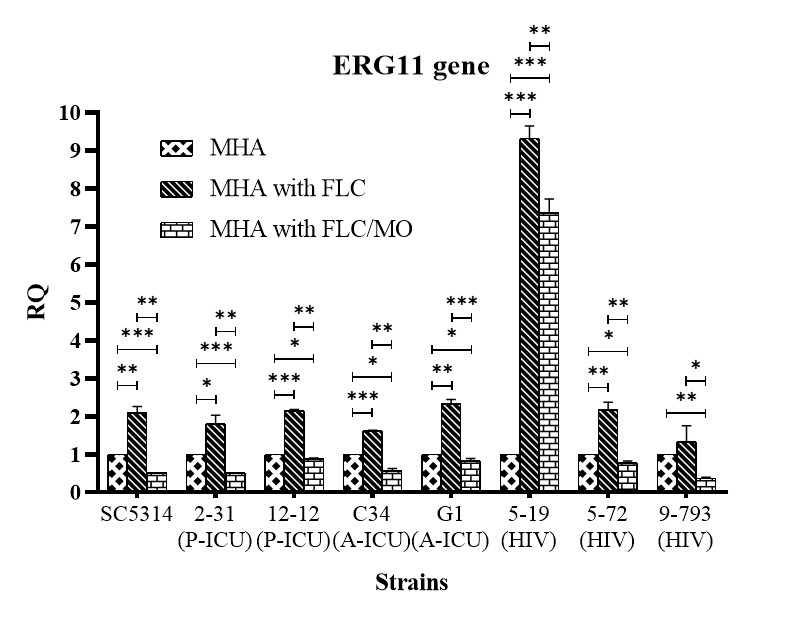


**Supplemental figure 3. Relative *ERG11* transcription in *Candida albicans* strains across antifungal treatment conditions.**

*ERG11* mRNA abundance was quantified by RT-PCR and normalized to a constitutively expressed reference gene 18S rRNA. Clinical and reference isolates were grown on Mueller–Hinton agar (MHA), MHA supplemented with fluconazole (FLC), or MHA co-supplemented with FLC and milbemycin oxime (MO). Relative quantification (RQ) was derived using the 2^(–ΔΔCt) method. Statistical comparisons were conducted on values using two-tailed t-tests. Data represent mean ± SD from three independent biological replicates. Significance is denoted as: ns, p > 0.05; *, p < 0.05; **, p < 0.01; ***, p < 0.001.
