# Supplementary material for: Genetic variation and mutational determinants of azole resistance in Candida albicans strains of oropharyngeal colonization in HIV patients and bloodstream infections
Source: J Biomed Sci. 2026 Feb 22;33:20. doi: 10.1186/s12929-026-01231-4 (PMC12925357; doi:10.1186/s12929-026-01231-4)
Supplement: Supplementary file 4 — Additional file 4. [file 12929_2026_1231_MOESM4_ESM.doc]

Supplemental Table 1. Summary of *Erg11* alterations in nucleotide and amino acid sequences detected in azole-resistant *C. albicans* isolates.

| Species  Sources | Isolates | MIC (mg/L) | | | | Mutated sites in ERG11 gene | Substitution of amino acid |
| --- | --- | --- | --- | --- | --- | --- | --- |
|  |  | FLC | VOC | ITC | POS |  |  |
| Pediatric patients with *Candida* BSIs | 2-31 | **8.0** | **0.5** | **0.25** | **0.12** | T394C, G1343A, G1570A | Y132H, G448E, E524K |
|  | 2-34 | **16.0** | **2.0** | **0.5** | **0.12** | T394C, G1343A, G1570A | Y132H, G448E, E524K |
|  | 2-44 | **16.0** | **2.0** | **0.5** | **0.12** | T394C, G1343A, G1570A | Y132H, G448E, E524K |
|  | 12-12 | **8.0** | **2.0** | **0.5** | **0.25** | T769C, G1570A | Y257H, E524K |
|  | 15-01 | **16.0** | **1.0** | **0.5** | **0.25** | T394C, G1343A, G1570A | Y132H, G448E, E524K |
|  | 15-02 | **16.0** | **1.0** | **0.5** | **0.25** | T394C, G1343A, G1570A | Y132H, G448E, E524K |
| Adult patients with *Candida* BSIs | C01 | **16.0** | **0.25** | **0.5** | **1.0** | A798C | E266D |
|  | D34 | **16.0** | **0.5** | **0.5** | **1.0** | T348A, T459G | D116E, D153E |
|  | F01 | **8.0** | **0.25** | **1.0** | **2.0** | T769C, | Y257H |
|  | G01 | **16.0** | **1.0** | **1.0** | **1.0** | G340T, T769C, | A114S,Y257H |
|  | G02 | **16.0** | **1.0** | **0.25** | **0.5** | T394C, G1343A, G1570A | Y132H, G448E, E524K |
| Colonized in HIV patients | 19 | **128.0** | **8.0** | **16.0** | **8.0** | T348A, T459G, A798C | D116E, D153E, E266D |
|  | 72 | **32.0** | **8.0** | **16.0** | **8.0** | T433C | F145L |
|  | 96 | 0.5 | 0.015 | **0.12** | 0.06 | A798C, G1309A | E266D, V437I |
|  | 174 | 2.0 | 0.03 | **0.25** | **0.12** | A798C, G1462A | E266D,V488I |
|  | 357 | 0.5 | 0.015 | **0.12** | **0.12** | T348A, T459G | D116E, D153E |
|  | 364 | 1.0 | 0.03 | **0.12** | 0.06 | X | X |
|  | 400 | **256.0** | **8.0** | **16.0** | **8.0** | T348A, T459G, A798C | D116E, D153E, E266D |
|  | 422 | **64.0** | **8.0** | **16.0** | **8.0** | T348A, A383C | D116E, K128T |
|  | 509 | **256.0** | **8.0** | **16.0** | **8.0** | T348A, T459G, A798C | D116E, D153E, E266D |
|  | 565 | 1.0 | 0.015 | **0.12** | **0.12** | A798C, G1309A | E266D, V437I |
|  | 391 | 0.5 | <0.008 | **0.12** | 0.03 | G1462A | V488I |
|  | 515 | 2.0 | 0.06 | **0.25** | **0.12** | T348A, T459G | D116E, D153E |
|  | 793 | 0.5 | 0.03 | **0.25** | **0.12** | T348A, T459G, A798C | D116E, D153E, E266D |
|  | 829 | **16.0** | **4.0** | **16.0** | **8.0** | T348A, T459G | D116E, D153E |
|  | 1222 | 1.0 | 0.06 | **0.25** | **0.12** | T348A, T459G | D116E, D153E |
|  | 1321 | 0.5 | 0.015 | **0.12** | 0.06 | T348A, A798C, C799T | D116E, E266D , R267C |

MIC: minimal inhibitory concentration; ITC: itraconazole; VOC: voriconazole; POS: posaconazole; FLC: fluconazole; a: new silent point mutation; b: new point mutation; In bold: resistant strains based on the EUCAST and CLSI Antifungal Clinical Breakpoints.
