# Supplementary material for: Genetic variation and mutational determinants of azole resistance in Candida albicans strains of oropharyngeal colonization in HIV patients and bloodstream infections
Source: J Biomed Sci. 2026 Feb 22;33:20. doi: 10.1186/s12929-026-01231-4 (PMC12925357; doi:10.1186/s12929-026-01231-4)
Supplement: Supplementary file 5 — Additional file 5. [file 12929_2026_1231_MOESM5_ESM.doc]

**Supplemental Table 2. Missense mutations identified in the *CDR1* genes of the *C. albicans* isolates from ICU and HIV patients^$^.**

| **[2a] Cdr1 (aa)** | | **350** | **368** | **372** | **388** | **396** | **427** | **441** | **468** | **842** | **916** | **948** | **949** | **950** | **993** | **1049** | **1071** | **1416** |
| --- | --- | --- | --- | --- | --- | --- | --- | --- | --- | --- | --- | --- | --- | --- | --- | --- | --- | --- |
| **SC5314** | | V/V | D/D | K/K | T/T | K/K | Y/Y | Y/Y | R/R | T/T | I/I | E/E | T/T | T/T | L/L | D/D | R/R | E/E |
| **Pediatric ICU** | **2-31** | - | - | - | - | - | - | - | - | - | - | E/P | - | T/S | - | - | - | - |
|  | **12-12** | - | - | - | - | - | - | - | - | - | - | E/P | - | - | - | - | - | - |
| **Adult ICU** | **C34** | - | - | - | - | - | - | - | - | - | V/V | P/P | - | S/S | - | - | - | - |
|  | **G1** | - | - | - | - | - | - | - | - | - | - | - | T/P | T/S | - | - | - | - |
| **HIV  Oral** | **5-19** | - | - | - | - | - | - | - | - | **S/S** | V/V | P/P | - | S/S | - | - | - | - |
|  | **5-72** | - | - | - | - | - | - | - | - | **S/S** | V/V | P/P | - | S/S | - | - | - | - |
|  | **9-793** | - | - | - | - | - | Y/F | - | - | **S/S** | V/V | P/P | - | S/S | - | - | - | E/A |

| **[2b] Cdr2 (aa)** | | **21** | **50** | **59** | **92** | **111** | **112** | **121** | **122** | **131** | **179** | **348** | **367** | **371** | **439** | **466** |
| --- | --- | --- | --- | --- | --- | --- | --- | --- | --- | --- | --- | --- | --- | --- | --- | --- |
| **SC5314** | | S/S | G/G | A/A | E/E | S/S | D/D | V/V | A/A | I/I | E/E | T/T | E/E | N/N | F/F | G/G |
| **Pediatric ICU** | **2-31** | - | - | A/T | - | - | - | - | - | - | - | - | - | N/K | F/Y |  |
|  | **12-12** | - | - | - | - | - | - | - | - | - | - | T/V | E/D | N/K | F/Y |  |
| **Adult ICU** | **C34** | - | - | - | - | - | - | - | - | - | - | - | - | - | - | G/R |
|  | **G1** | - | - | A/T | - | - | - | - | - | - | E/K | T/V | E/D | N/K | F/Y | - |
| **HIV  Oral** | **5-19** | - | - | A/T | E/K | S/P | D/E | **V/I** | A/G | I/V | E/K | - | - | - | F/Y | G/R |
|  | **5-72** | - | - | - | - | - | - | **V/I** | - | - | - | - | - | - | - | - |
|  | **9-793** | - | G/D | A/T | - | S/P | D/E | **V/I** | A/G | I/V | E/K | - | - | - | F/Y | G/R |
| **(aa)** | | **484** | **620** | **632** | **683** | **946** | **948** | **1069** | **1075** | **1091** | **1094** | **1305** | **1337** | **1473** | **1474** | **1497** |
| **SC5314** | | S/S | A/A | S/S | R/R | E/E | T/T | K/K | K/K | T/T | N/N | P/P | L/L | G/G | I/I | M/M |
| **Pediatric ICU** | **2-31** | - | - | - | - | - | - | - | - | - | - | P/L | - | - | - | - |
|  | **12-12** | - | A/T | - | R/K | - | - | - | - | - | - | P/L | L/F | G/A | I/V | M/L |
| **Adult ICU** | **C34** | - | - | - | - | - | - | - | - | - | - | - | - | - |  | - |
|  | **G1** | - | A/T | - | R/K | - | - | - | - | - | - | P/L | L/F | G/A | I/V | M/L |
| **HIV  Oral** | **5-19** | S/A | - | - | - | - | - | **R/R** | K/R | T/I | N/S | - | **F/F** | G/A | I/V | M/L |
|  | **5-72** | - | - | - | R/K | - | - | **K/R** | - | - | - | - | **F/F** | - | - | L/L |
|  | **9-793** | S/A | - | - | - | - | - | **R/R** | K/R | T/I | N/S | - | **F/F** | G/A | I/V | M/L |

$: Protein-coding sequences of *Candida albicans* (taxid: 5476) were retrieved from the NCBI nucleotide database (nt/nt) and analyzed using the tBLASTx algorithm. Low-frequency amino acid polymorphisms (less than 5%) were filtered out. Cells shaded in gray indicate mutations observed in the genome-sequenced strains of this study, while cells with a white background represent missense mutation sites obtained from the NCBI database. Two alleles coding for amino acids are separated by a slash. The IUPAC amino acid code is used to denote the substitutions. A dash ("-") indicates no change in the amino acid sequence.
