# Supplementary material for: Genetic variation and mutational determinants of azole resistance in Candida albicans strains of oropharyngeal colonization in HIV patients and bloodstream infections
Source: J Biomed Sci. 2026 Feb 22;33:20. doi: 10.1186/s12929-026-01231-4 (PMC12925357; doi:10.1186/s12929-026-01231-4)
Supplement: Supplementary file 6 — Additional file 6. [file 12929_2026_1231_MOESM6_ESM.doc]

**Supplementary Table 3a. Missense mutations identified in the *ERG11* genes of the *C. albicans* isolates from the ICU and HIV patients^$^.**

| **Erg11 (aa)** | | **114** | **116** | **128** | **132** | **145** | **153** | **257** | **263** | **266** | **276** | **437** | **449** | **456** | **467** | **488** |
| --- | --- | --- | --- | --- | --- | --- | --- | --- | --- | --- | --- | --- | --- | --- | --- | --- |
| **SC5314** | | A/A | D/E | K/T | Y/Y | F/F | D/D | Y/Y | S/S | E/E | L/L | V/V | F/F | V/V | R/R | V/V |
| **Pediatric ICU** | **2-31** | - | D/D | - | H/H | - | - | - | - | - | - | - | E/E | - | - | - |
|  | **12-12** | S/S | D/D | - | - | - | - | H/H | - | - | - | - | - | - | - | - |
| **Adult ICU** | **C34** | - | - | - | - | - | D/E | - | - | - | - | - | - | - | - | - |
|  | **G1** | S/S | D/D | - | - | - | - | H/H | - | - | - | - | - | - | - | - |
| **HIV  Oral** | **5-19** | - | - | - | - | - | E/E | - | - | D/D | - | - | - | - | - | - |
|  | **5-72** | - | - | - | - | F/L | - | - | - | - | - | - | - | - | - | - |
|  | **9-793** | - | - | - | - | - | E/E | - | - | D/D | - | - | - | - | - | - |

$: Protein-coding sequences of *Candida albicans* (taxid: 5476) were retrieved from the NCBI nucleotide database (nt/nt) and analyzed using the tBLASTx algorithm. Low-frequency amino acid polymorphisms (less than 5%) were filtered out. Cells shaded in gray indicate mutations observed in the genome-sequenced strains of this study, while cells with a white background represent missense mutation sites obtained from the NCBI database. Two alleles coding for amino acids are separated by a slash. The IUPAC amino acid code is used to denote the substitutions. A dash ("-") indicates no change in the amino acid sequence.

**Supplementary Table 3b. Missense mutations identified in the *UPC2* genes of the *C. albicans* isolates from ICU and HIV patients**

| **Upc2 (aa)** | | **68** | **142** | **228** | **232** | **273** | **303** | **426** | **544** |
| --- | --- | --- | --- | --- | --- | --- | --- | --- | --- |
| **SC5314** | | R/R | I/I | S/S | S/S | T/T | S/S | G/G | A/A |
| **Pediatric ICU** | **2-31** | - | - | - | - | - | - | - | - |
|  | **12-12** | - | - | - | - | - | - | - | - |
| **Adult ICU** | **C34** | - | I/S | - | - | T/A | - | - | - |
|  | **G1** | - | - | - | - | - | - | - | A/V |
| **HIV  Oral** | **5-19** | R/K | - | S/N | - | - | - | - | - |
|  | **5-72** | - | S/S | - | - | - | - | - | - |
|  | **9-793** | R/K | I/S | S/N | - | T/A | - | G/E | - |

**Supplementary Table 4a. Missense mutations identified in the *TAC1* genes of the *C. albicans* isolates of ICU and HIV patients.**

| **Tac1 (aa)** | | **4** | **6** | **39** | **47** | **94** | **104** | **131** | **153** | **189** | **199** | **206** | **207** | **269** | **396** | **568** | **736** | **764** | **772** | **776** | **829** | **896** | **935** | **941** |
| --- | --- | --- | --- | --- | --- | --- | --- | --- | --- | --- | --- | --- | --- | --- | --- | --- | --- | --- | --- | --- | --- | --- | --- | --- |
| **SC5314** | | S/S | S/S | A/A | K/K | G/G | F/V | L/L | S/S | F/F | S/N | R/H | V/A | Y/Y | N/S | S/S | A/A | S/S | N/K | D/N | E/Q | N/N | S/L | S/P |
| **Pediatric ICU** | **2-31** | - | - | - | - | - | - | - | - | - | - | - | - | - | - | - | - | - | - | - | - | - | - | - |
|  | **12-12** | - | - | - | - | - | F/V | - | - | - | - | R/H | - | - | - | - | A/V | - | N/K | D/N | - | - | S/L | L/P |
| **Adult ICU** | **C34** | - | - | - | - | - | V/V | - | - | - | N/N | H/H | A/A | - | S/S | - | - | - | - | N/N | E/Q | N/S | - | P/P |
|  | **G1** | - | - | - | - | - | F/V | - | - | - | - | - | - | - | - | - | V/V | - | K/K | N/N | E/Q | - | - | L/P |
| **HIV  Oral** | **5-19** | - | - | - | - | - | V/V | - | - | - | S/N | R/H | V/A | - | N/S | - | - | S/N | - | D/N | E/Q | N/S | - | L/P |
|  | **5-72** | - | - | A/T | - | G/R | - | - | - | - | - | R/H | V/A | - | - | - | - | - | - | - | - | - | - | - |
|  | **9-793** | - | - | - | - | - | V/V | - | - | - | S/N | R/H | V/A | - | N/S | - | - | S/N | - | D/N | E/Q | N/S | - | L/P |

**Supplementary Table 4b. Missense mutations identified in the *NDT80* genes of the *C. albicans* isolates of ICU and HIV patients**

| **Ndt80 (aa)** | | **22** | **142** | **160** | **313** |
| --- | --- | --- | --- | --- | --- |
| **SC5314** | | S/S | A/A | Q/Q | S/S |
| **Pediatric ICU** | **2-31** | - | - | - | - |
|  | **12-12** | - | - | - | - |
| **Adult ICU** | **C34** | - | - | - | - |
|  | **G1** | - | - | - | - |
| **HIV  Oral** | **5-19** | - | - | - | - |
|  | **5-72** | P/P | - | - | - |
|  | **9-793** | - | A/T | Q/H | - |

**Supplementary Table 5a. Missense mutations identified in the *MDR1* genes of the *C. albicans* isolates from ICU and HIV patients.**

| **Mdr1 (aa)** | | **32** | **35** | **92** | **152** | **239** | **313** | **317** | **325** |
| --- | --- | --- | --- | --- | --- | --- | --- | --- | --- |
| **SC5314** | | D/D | V/V | D/D | V/V | W/W | I/I | D/D | V/I |
| **Pediatric ICU** | **2-31** | - | - | - | - | - | - | - | - |
|  | **12-12** | - | - | - | - | - | - | - | - |
| **Adult ICU** | **C34** | - | - | - | V/I | W/* | - | - | V/I |
|  | **G1** | - | - | - | - | - | - | - | I/I |
| **HIV  Oral** | **5-19** | - | - | - | - | - | - | - | V/I |
|  | **5-72** | - | - | - | - | - | - | - | V/I |
|  | **9-793** | - | - | - | - | - | - | - | V/I |

**Supplementary Table 5b. Missense mutations identified in the *MRR1* genes of *C. albicans* isolates from the ICU and HIV patients.**

| **Mrr1**  **(aa)** | | **19** | **27** | **60** | **72** | **75** | **102** | **107** | **171** | **191** | **248** | **341** | **406** | **409** | **680** | **683** | **687** | **706** | **844** | **950** | **1020** | **1032** | **1037** | **1045** | **1056** |
| --- | --- | --- | --- | --- | --- | --- | --- | --- | --- | --- | --- | --- | --- | --- | --- | --- | --- | --- | --- | --- | --- | --- | --- | --- | --- |
| **SC5314** | | P/P | V/V | V/V | S/S | G/G | A/A | S/S | S/S | S/S | L/L | V/V | S/S | G/G | S/S | P/P | S/S | S/S | S/S | S/S | E/E | F/F | S/S | S/S | S/S |
| **Pediatric ICU** | **2-31** | - | - | - | - | - | - | - | - | - | - | V/E | - | G/E | - | - | - | - | - | - | E/Q | - | - | - | - |
|  | **12-12** | - | - | - | - | - | - | - | - | - | - | - | - | - | - | - | - | - | - | - | E/Q | - | - | - | - |
| **Adult ICU** | **C34** | - | - | - | - | - | - | - | - | - | - | - | - | - | - | - | - | - | - | - | E/Q | - | - | - | - |
|  | **G1** | - | - | - | - | - | - | - | - | - | L/V | V/E | - | - | - | - | - | - | - | - | E/Q | - | - | - | - |
| **HIV  Oral** | **5-19** | P/L | - | - | - | G/R | A/T | - | - | - | - | - | - | - | - | - | - | - | - | - | Q/Q | - | S/L | - | - |
|  | **5-72** | - | - | - | - | - | - | - | - | - | - | - | - | - | - | - | - | - | - | - | - | - | - | - | - |
|  | **9-793** | P/L | - | V/A | - | G/R | A/T | - | - | - | - | - | - | - | - | - | - | - | - | - | Q/Q | F/L | S/L | - | - |
